# Phylogeny of *Drosophila saltans* group (Diptera: Drosophilidae) based on morphological and molecular evidence

**DOI:** 10.1371/journal.pone.0266710

**Published:** 2022-04-07

**Authors:** Bruna Emilia Roman, Diego J. Santana, Carolina Prediger, Lilian Madi-Ravazzi

**Affiliations:** 1 Departament of Biology, Institute of Biosciences, Humanities and Exact Sciences, São Paulo State University (UNESP), São José do Rio Preto, São Paulo, Brazil; 2 Biosciences Institute, Federal University of Mato Grosso do Sul, Campo Grande, Mato Grosso do Sul, Brazil; Laboratoire de Biologie du Développement de Villefranche-sur-Mer, FRANCE

## Abstract

*Drosophila saltans* group belongs to the subgenus *Sophophora* (family Drosophilidae), and it is subdivided into five subgroups, with 23 species. The species in this group are widely distributed in the Americas, primarily in the Neotropics. In the literature, the phylogenetic reconstruction of this group has been performed with various markers, but many inconsistencies remain. Here, we present a phylogenetic reconstruction of the *saltans* group with a greater number of species, 16 species, which is the most complete to date for the *saltans* group and includes all subgroups, in a combined analysis with morphological and molecular markers. We incorporated 48 morphological characters of male terminalia, the highest number used to date, and molecular markers based on mitochondrial genes *COI* and *COII*. Based on the results, which have recovered the five subgroups as distinct lineages, we propose a new hypothesis regarding the phylogenetic relationships among the subgroups of the *saltans* group. The relationships of the species within the *sturtevanti* and *elliptica* subgroups were well supported. The *saltans* subgroup showed several polytomies, but the relationship between the sibling species *D*. *austrosaltans* and *D*. *saltans* and their close relation with *D*. *nigrosaltans* were well supported in the molecular and total evidence analyses. The morphological analysis additionally supported the formation of the clade *D*. *nigrosaltans*—*D*. *pseudosaltans*. The observed polytomies may represent synchronous radiations or have resulted from speciation rates that have been too fast relative to the pace of substitution accumulation.

## Introduction

Phylogenetic reconstructions based on integrative analyses of different sets of characteristics (e.g., molecular and morphological characters) enable us to deduce robust evolutionary hypotheses [1, 2]. In addition, approaches that use several lines of evidence can reconstruct better relationships among taxa, mainly within groups with historical problems [3, 4]. Although molecular methods have more often been used to infer phylogenetic relationships between organisms, the use of nonmolecular data is still highly recommended for identifying synapomorphies [5]. Morphological data are thus fundamental for decision-making in taxonomy and systematics [6]. Considering the genus *Drosophila*, the use of adult terminalia characters is particularly useful because they are the least homoplastic [7]. Among the various groups that need more robust phylogenetic evaluation, *Drosophila* species from the Neotropical region need a more complete and better supported phylogenetic hypothesis [8].

*Drosophila saltans* group is nested within the genus *Drosophila* and subgenus *Sophophora* and is closely related to the *willistoni* sister clade in the New World *Drosophila* diversification [9]. The geographical distribution of species in the *saltans* group extends across the entire region of Mexico to the state of Rio Grande do Sul in southern Brazil [10–13]. The *parasaltans* and *cordata* subgroups occur only in the Neotropical region, while some species of the *elliptica*, *sturtevanti* and *saltans* subgroups occur in the Neotropical and Nearctic regions [10].

Historically, the *saltans* group was first divided into two subgroups [14], but this division was later modified by Pavan and Magalhães [15] and Magalhães [16]. After that, Magalhães and Björnberg [17] divided the group into five subgroups, without naming, based on morphological characters with an emphasis on male terminalia. Then, with a total of 19 species, the five subgroups were named *saltans* (*D*. *saltans*, *D*. *austrosaltans*, *D*. *lusaltans*, *D*. *prosaltans*, *D*. *nigrosaltans*, *D*. *pseudosaltans* and *D*. *septentriosaltans*), *sturtevanti* (*D*. *sturtevanti*, *D*. *milleri* and *D*. *rectangularis*), *parasaltans* (*D*. *parasaltans*, *D*. *subsaltans* and *D*. *pulchella*), *elliptica* (*D*. *elliptica*, *D*. *emarginata*, *D*. *neoelliptica* and *D*. *neosaltans*) and *cordata* (*D*. *cordata* and *D*. *neocordata*) [10]. However, Vilela and Bächli [18] observed the lectotype of *D*. *pulchella* and inserted this species into the *sturtevanti* subgroup and even suggested it as a synonym to *D*. *sturtevanti*. Mourão and Bicudo [11] added two new species to the *sturtevanti* subgroup (*D*. *dacunhai* and *D*. *magalhaesi*). Recently, Guillin and Rafael [12] introduced the species *D*. *neoprosaltans* in this group, and Madi-Ravazzi et al. [19] included another new species (*D*. *lehrmanae*) in the *sturtevanti* subgroup. Throughout these investigations, the number of species belonging to the *saltans* group increased to 23 [20].

Many studies have discussed the phylogeny of the *saltans* group [21–29]. However, some were more relevant regarding the robustness of the analysis and support of the trees. O’Grady et al. [24] performed the first molecular phylogeny with nine species of the *saltans* group. According to their total evidence tree, the *cordata* subgroup would have branched off the earliest, followed by the *elliptica* subgroup (*D*. *emarginata*), and the *sturtevanti* subgroup (*D*. *milleri* and *D*. *sturtevanti*) was recovered as the sister of the *saltans-parasaltans* clade. However, the relationships among species of the *saltans* subgroup were not well resolved, suggesting a recent divergence [24]. Moreover, Rodríguez-Trelles et al. [25] also proposed a phylogeny of the *saltans* group based on analyses of some molecular markers including *xanthine dehydrogenase* (*Xdh*). One of the trees generated with robust support showed the *parasaltans* subgroup (*D*. *subsaltans*) as sister to all others, followed by branching off the *sturtevanti* (*D*. *sturtevanti*), *elliptica* (*D*. *emarginata*), *cordata* (*D*. *neocordata*) and *saltans* (*D*. *saltans* and *D*. *prosaltans*) subgroups. Thus, the differences in marker genes and ingroup taxon-sampling between these studies would have affected the phylogenetic inference, resulting in topologies differing in the basal branch.

Morphological characters have also been fundamental tools in the delineation of the *saltans* phylogeny. Yassin [28] inferred the phylogeny of this group by coding 40 morphological characters from different life stages and of external and internal morphology. The generated tree supported the *sturtevanti* subgroup (*D*. *sturtevanti* and *D*. *milleri*) as the sister of all others in the group. The other subgroups formed two clades, one consisting of the *cordata* (*D*. *neocordata*) and *elliptica* (*D*. *emarginata*) subgroups and the other consisting of the *parasaltans* (*D*. *subsaltans*) and *saltans* (*D*. *saltans*, *D*. *prosaltans*, *D*. *austrosaltans* and *D*. *lusaltans*) subgroups. Again, the relationships between *saltans* subgroup species were not resolved [28]. Last, Souza et al. [29] used morphological data from male terminalia to infer the phylogeny of the group. This work demonstrated the *cordata* (*D*. *neocordata*) subgroup as the sister taxon of all others and the formation of two large clades, one consisting of the *elliptica* (*D*. *emarginata*) and *sturtevanti* (*D*. *sturtevanti*, *D*. *dacunhai* and *D*. *milleri)* subgroups and the other of the *parasaltans* (*D*. *parasaltans*) and *saltans* (*D*. *saltans*, *D*. *prosaltans*, *D*. *lusaltans* and *D*. *austrosaltans)* subgroups [29].

The studies mentioned above used a limited number of taxa and few male terminalia characters, which are the most variable even among closely related species and mainly used to distinguish species in insect taxonomy, in their phylogenetic reconstructions, resulting in inconsistencies mainly for the *saltans* subgroup. Yassin [28] analyzed the inconsistencies of molecular phylogenetic inferences for the *saltans* group and pointed out that codon usage bias (CUB) may be an issue in this clade because nonstationarity and nonhomogeneity of the nucleotide composition can distort phylogenetic inferences, when compositional changes do not occur according to the genealogy of the species. Indeed, the Neotropical *Sophophora* (i.e., the *saltans* and *willistoni* groups) have higher frequencies of adenine and thymine at the third position of the code of their nuclear genome [25, 30–32]. A study of the nuclear and mitochondrial CUB patterns in other insects pointed out that the mitochondrial genome has higher CUB [33]. Therefore, we present a new hypothesis for phylogenetic relationships among subgroups of the *saltans* group based mainly on a total evidence dataset (morphological and molecular markers) and a greater number of species (16 species) than that already employed. In addition, we explored some difficulties found in the phylogenetic reconstruction of this group, focusing on the *saltans* subgroup.

## Materials and methods

### Taxon sampling

In the present study, 16 of the 23 species of the *saltans* group were evaluated based on morphological and molecular data. The species, strains and geographical origin are listed in S1 Table.

### Terminalia preparation and morphological characters

The structures of terminalia were dissected and mounted based on Kaneshiro’s [34] technique. The distal two-thirds of the abdomen of each fly was extracted by stylets and placed in a microtube containing 10% KOH solution for 15 minutes in a water bath; then, the structures were transferred to a microtube containing a drop of eugenol and incubated for 24 h. After that, the terminalia was dissected with the aid of a stereomicroscope and stylets. The structures were dehydrated with pure acetone and mounted on stubs with copper tape for adhesion and electron conductivity [29]. The samples were sputter coated with gold in and analyzed by scanning electron microscopy (LEO 435 VPi Zeiss).

Based on the descriptions and analysis of the male terminalia electron micrographs by Roman and Madi-Ravazzi [35], 48 morphological characters were collected, and a matrix was coded in absence (0), presence (1) and presence with modifications (2) (Table 1).

**Table 1:** Matrix of 48 coded morphological characters.

	*D*. *austrosaltans*	*D*. *lusaltans*	*D*. *nigrosaltans*	*D*. *prosaltans*	*D*. *pseudosaltans*	*D*. *saltans*	*D*. *septentriosaltans*	*D*. *dacunhai*	*D*. *milleri*	*D*. *sturtevanti*	*D*. *lehrmanae*	*D*. *emarginata*	*D*. *neoelliptica*	*D*. *neosaltans*	*D*. *neocordata*	*D*. *parasaltans*	*D*. *willistoni*
0. Epandrial Ventral Process	0	0	0	0	0	0	0	1	1	1	1	1	1	0	0	1	0
1. Semi-elliptical shaped surstylus	1	1	1	1	1	1	1	0	0	0	0	0	0	0	0	0	0
2. Hand-shaped surstylus	0	0	0	0	0	0	0	0	0	0	0	0	0	0	0	1	0
3. Elongated surstylus	0	0	0	0	0	0	0	0	0	0	0	1	1	1	0	0	0
4. Surstylar teeth arranged throughout the intern portion of the surstylus	1	1	1	1	1	1	1	0	0	0	0	1	1	1	0	0	0
5. Surstylar process	0	0	0	0	0	0	0	0	0	0	0	0	0	0	1	0	0
6. Elongated hypandrium	1	1	1	1	1	1	1	0	0	0	0	2	2	2	0	1	0
7. Median gonocoxites rounded and convergent	0	1	0	0	0	0	0	0	0	0	0	0	0	0	0	0	0
8. Long phallapodeme	1	1	1	1	1	1	1	0	0	0	0	0	1	1	0	1	0
9. Sickle-shaped aedeagus	0	0	0	0	0	0	0	0	0	0	0	1	1	0	0	0	0
10. Pregonites fused to the end	0	0	0	0	0	0	0	0	0	0	0	1	1	0	0	0	0
11. Pregonites fused into a single structure	0	0	0	0	0	0	0	0	0	0	0	0	0	0	1	0	0
12. Long and bifurcate ventral postgonites	0	0	0	0	0	0	0	0	0	0	0	0	0	0	1	0	0
13. Aedeagus apex with punctiform projection	0	0	0	0	0	0	0	1	1	1	1	0	0	0	0	0	0
14. Short punctiform projection at the aedeagal apex	0	0	0	0	0	0	0	0	0	0	1	0	0	0	0	0	0
15. Bipartite aedeagal apex	0	0	0	0	0	0	0	0	0	0	0	1	1	0	0	0	0
16. Cylindrical aedeagal apex	0	0	0	0	0	0	0	0	0	0	0	0	0	1	0	0	0
17. Membranous aedeagal apex	0	0	0	0	0	0	0	0	0	0	0	0	0	0	0	1	0
18. Frontal region of aedeagal apex with a pair of chitinous hooks	0	0	0	0	0	0	0	0	0	0	0	0	0	0	1	0	0
19. Apical crest covered with scales at the aedeagal apex	1	0	0	0	0	0	0	0	0	0	0	0	0	0	0	0	0
20. Groove in the aedeagal apex	0	1	0	0	0	0	0	0	0	0	0	0	0	0	0	0	0
21. Bristles at the aedeagal apex	0	1	1	0	1	1	0	0	0	0	0	0	0	0	0	0	0
22. Scales at the aedeagal apex	1	0	1	1	1	0	1	0	0	0	0	0	0	0	0	0	0
23. Elongated and curved back aedeagal apex	0	0	1	0	1	0	0	0	0	0	0	0	0	0	0	0	0
24. Aedeagal sheath	1	1	1	1	1	1	1	0	0	0	0	0	0	0	0	1	0
25. Smooth aedeagal sheath with serrated edge	0	1	0	0	0	1	0	0	0	0	0	0	0	0	0	1	0
26. Spicules-like structures in the dorsal region of aedeagal sheath	0	0	0	1	0	0	0	0	0	0	0	0	0	0	0	0	0
27. Serrated crests on the aedeagal sheath	1	0	0	0	0	0	1	0	0	0	0	0	0	0	0	0	0
28. Ventral postgonites	1	1	1	1	1	1	1	1	1	1	1	0	0	0	0	1	0
29. Aedeagus with only one ventral postgonite	0	0	0	0	0	0	0	1	1	1	1	0	0	0	0	0	0
30. A pair of ventral postgonites	1	1	1	1	1	1	1	0	0	0	0	0	0	0	0	1	0
31. A pair of bifurcate ventral postgonites	0	0	0	0	0	0	0	0	0	0	0	0	0	0	0	1	0
32. Scales in the upper ventral region of aedeagus	0	0	0	0	0	0	0	1	1	1	1	0	0	0	0	0	0
33. Scales at the ventral postgonite	0	0	0	0	0	0	0	1	1	0	0	0	0	0	0	0	0
34. Enlarged ventral postgonite	0	0	0	0	0	0	0	0	1	0	0	0	0	0	0	0	0
35. Groove in the upper portion of ventral postgonite	0	0	0	0	0	0	0	1	1	0	0	0	0	0	0	0	0
36. Thin ventral postgonite without scales	0	0	0	0	0	0	0	0	0	1	1	0	0	0	0	0	0
37. Ventral postgonite slightly curved	0	0	0	0	0	0	0	1	0	0	0	0	0	0	0	0	0
38. Ventral postgonite combine with aedeagal apex result in a V-shaped	0	0	0	0	0	0	0	0	1	1	1	0	0	0	0	0	0
39. Ventral postgonite combine with aedeagal apex result in a C-shaped	0	0	0	0	0	0	0	1	0	0	0	0	0	0	0	0	0
40. A pair of lateral postgonites	0	0	0	0	0	0	0	0	0	0	0	1	1	1	0	0	0
41. A pair of aedeagal ventral process	1	1	1	1	1	1	1	0	0	0	0	0	0	0	0	0	0
42. Aedeagal ventral process bifurcated at the end	0	0	1	0	1	0	0	0	0	0	0	0	0	0	0	0	0
43. Enlarged and darkened aedeagal ventral process	0	0	0	0	1	0	0	0	0	0	0	0	0	0	0	0	0
44. Aedeagal ventral crest	0	0	0	0	0	0	0	0	0	0	0	0	1	1	0	0	0
45. Pair of aedeagal ventral crest	0	0	0	0	0	0	0	0	0	0	0	0	0	1	0	0	0
46. Cleft on the dorsal region of aedeagus	0	0	0	0	0	0	0	0	0	0	0	1	1	0	0	0	0
47. A pair of long protuberances arranged laterally and fused to the aedeagus	0	0	0	0	0	0	0	0	0	0	0	0	0	0	1	0	0

0 = absence, 1 = presence and 2 = presence with modifications.

### DNA sequencing

The genomic DNA of males of the species studied was extracted from the whole body and by individual maceration using a Wizard Genomic DNA Purification kit (Promega, Madison, WI, USA) following the manufacturer’s protocol.

The mitochondrial genes were amplified through polymerase chain reaction (PCR); the primer sequences and PCR annealing temperatures are shown in S2 Table. The amplification conditions were the same as in Madi-Ravazzi et al. [19]. Amplicons were sequenced by the Sanger method at Centro de Recursos Biológicos e Biologia Genômica (CREBIO). All generated sequences were deposited in GenBank (S1 Table).

### Parsimony analysis of morphological data

The analysis based on the maximum parsimony criterion was performed using the TNT [36] and Winclada software was used to visualize and edit trees [37]. The analysis was performed using heuristic searches by the traditional method: starting with the construction of Wagner trees and refining by branch swapping in the tree-bisection-reconnection (TBR) algorithm, with 1000 replicates and retaining 10 trees per replicate. The characters were analyzed with equal weights. A strict consensus was reached for the most parsimonious trees resulting. Branch support was estimated using bootstrap (BS) analysis [38] (significance values ≥ 70%), calculated with 1000 replicates, using the heuristic search and the traditional search methods.

### Molecular phylogenetic analysis

The editing of the sequences of the *COI* and *COII* genes as well as the comparison and creation of the consensus sequences of the forward and reverse sequences of each taxon were performed manually in BioEdit *7*.*2*.*5* [39], where they were also multiple aligned using ClustalW [40].

For phylogenetic analyses, we included *COI* and *COII* sequences (totaling 1,354 bp) from 16 species of the *saltans* group and *D*. *willistoni* as the outgroup (S1 Table). We determined the model of nucleotide substitution for each gene and the concatenated sequence datasets with jModelTest [41] using the Bayesian Information Criterion. The best-fit models were GTR+G. We performed a Bayesian phylogenetic analysis for the concatenated mitochondrial sequences (*COI* and *COII*) with MrBayes v3.2 [42]. We ran 100 million generations, sampling every 10,000 steps by using a Yule process tree prior. We checked for stationarity by visually inspecting trace plots and ensuring that all values for effective sample size (ESS) were above 200 in Tracer v.1.5 [43]. The first 25% of sampled genealogies were discarded as burn-in.

### Total-evidence analysis

We performed a total evidence analysis with both morphological and molecular data using partitioned Bayesian analyses of total evidence implemented in MrBayes v3.2 [42] to infer the phylogenetic relationships within the *saltans* group. This analysis assumes different models (e.g., θa, θb, θc) for different sets of partitions (e.g., Xa, Xb, Xc). The substitution models for *COI* (GTR+G) and *COII* (GTR+G) were tested in jModelTest [41] using the Bayesian information criterion. We used a gamma distribution for the morphological data [44]. We ran 100 million generations, sampling every 10,000 generations. We determined stationarity and ensured that all ESS values were 200 by using Tracer. The first 25% of sampled genealogies were discarded as burn-in.

### Codon usage bias

The GC content of the third codon position (GC3) and the relative synonymous codon usage (RSCU) for the concatenated sequences of *COI* and *COII* were calculated using the R package seqinR [45] and DNAsp [46], respectively. To evaluate the heterogeneity of the CUB for the *saltans* group, disparity index test [47] and composition distance were calculated in MegaX [48]. Two clustering methods were implemented using the averages RSCU: a hierarchical clustering generated by heatmap and a correspondence analysis were implemented in the R packages ggplot2 [49] and FactorMineR [50], respectively. In correspondence analysis, codons that were not seen for all species were removed (S3 Table).

## Results

**Fig 1:**
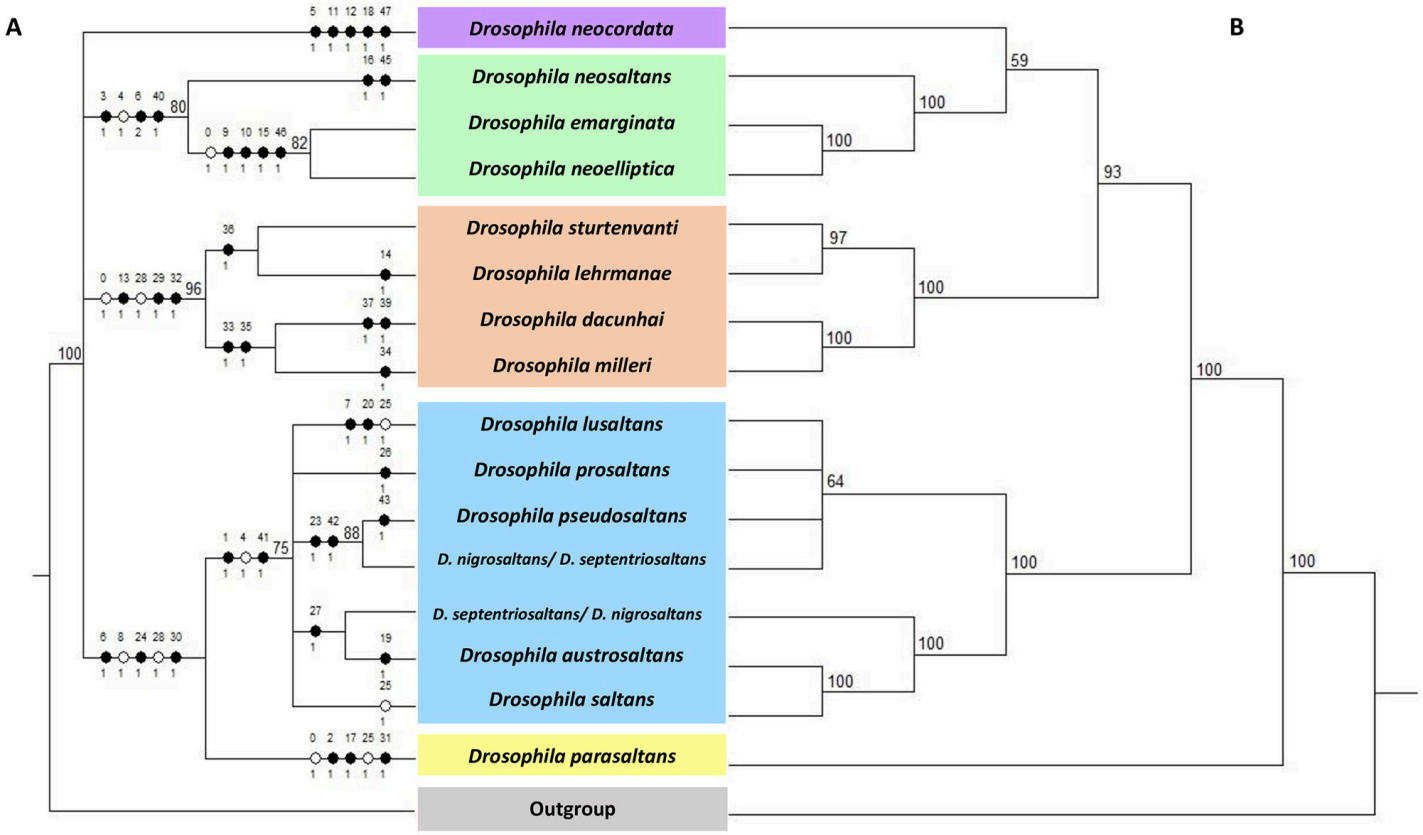
Comparison between morphological and molecular trees of the *saltans* group. (A) Phylogenetic relationships inferred by maximum parsimony based on morphological markers. Black circles correspond to nonhomoplastic apomorphies, and white circles correspond to homoplastic apomorphies, with character numbers above and state codes below (see Table 1). The bootstrap (BS) support values (≥ 70%) are shown above corresponding branches. (B) Phylogenetic relationships inferred by the analysis of the concatenated sequences of the mitochondrial markers *COI* and *COII*. Bayesian posterior probability (PP) is shown as percentage on each node.

Out of the 48 morphological characters coded, 29 were parsimony informative (i.e., shared by multiple species of the ingroup). The analysis resulted in eight equally parsimonious trees, and from these, the strict consensus was calculated (Fig 1A). The relationship among the subgroups was not resolved due to a polytomy. However, the species were grouped into the five subgroups [20]. The species of the *sturtevanti* subgroup were grouped in a clade with high support (BS = 96%) and supported by three synapomorphies: aedeagal apex with punctiform projection, aedeagus with only one ventral postgonite and scales in the upper ventral region of aedeagus. The species of the *elliptica* subgroup were grouped into one clade (BS = 80%), supported by synapomorphic characters of elongated surstylus and hypandrium and the presence of a pair of lateral postgonites. Within this clade, *D*. *emarginata* and *D*. *neoelliptica* formed an internal clade (BS = 82%). Although the *saltans* subgroup was recovered as a monophyletic group (BS = 75%), relationships among component species were less resolved, being left in a polytomy except for the sister relationship between *D*. *nigrosaltans* and *D*. *pseudosaltans* (BS = 88%). In this analysis, the *cordata* and *parasaltans* subgroups were each represented by a single species, *D*. *neocordata* and *D*. *parasaltans*, respectively. They were placed as independent branches with five and three autapomorphies, respectively: *D*. *neocordata* characterized by the presence of surstylus process, long and bifurcate ventral postgonites, pregonites basally fused into a single structure, frontal region of aedeagal apex with a pair of chitinous hooks, and a pair of long protuberances arranged laterally and fused to the aedeagus; and *D*. *parasaltans* by the presence of hand-shaped surstylus, membranous aedeagal apex, and the presence of a pair of bifurcate ventral postgonites (Table 1).

**Fig 2:**
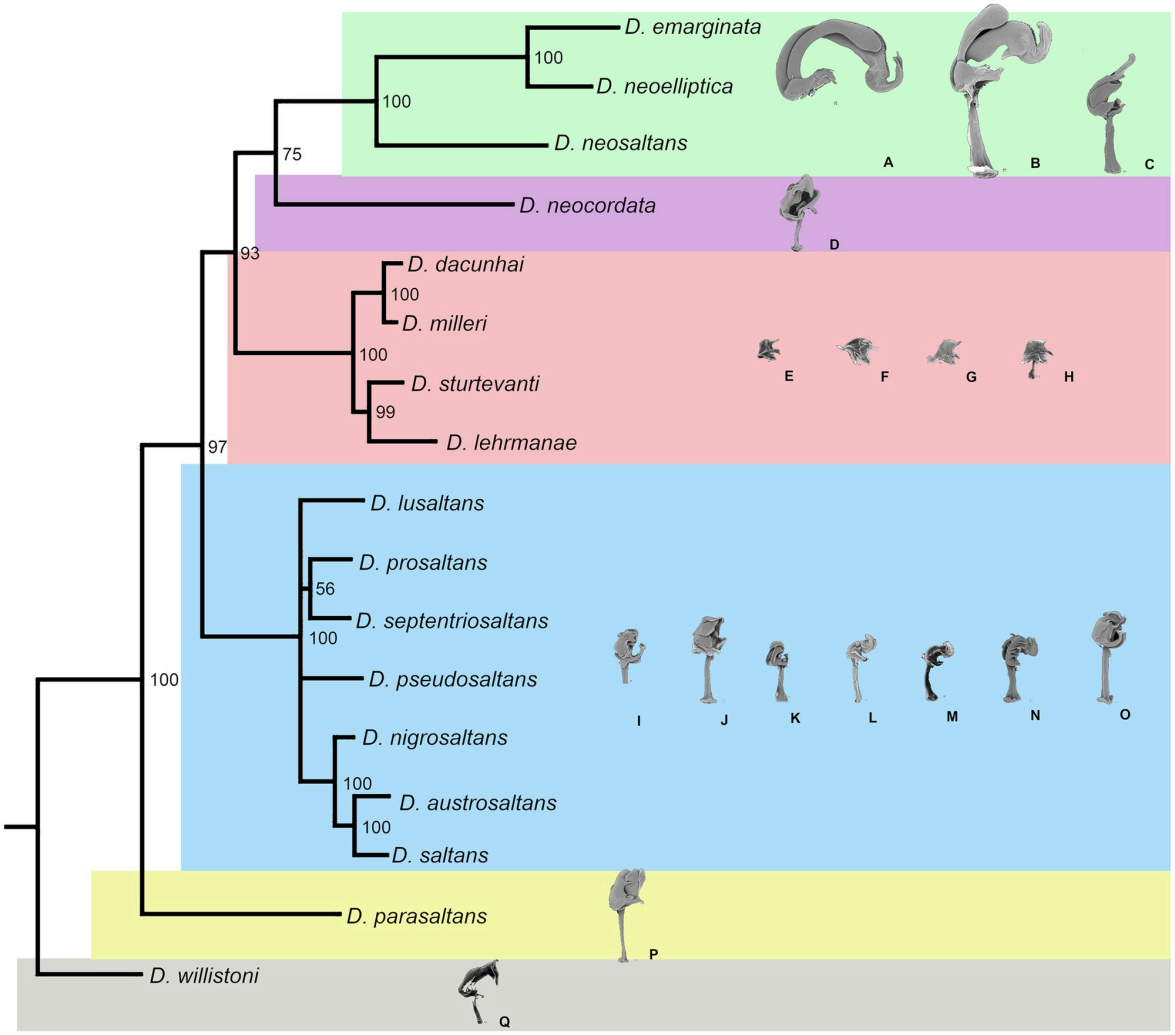
Phylogenetic relationships among species of the *saltans* group inferred by the total evidence analysis with morphological and molecular data. Bayesian posterior probability (PP) is shown as percentage on each node. The phallus of each species is shown in profile on the same scale. The letters represent the phallus of the following species: A = *D*. *emarginata*, B = *D*. *neoelliptica*, C = *D*. *neosaltans*, D = *D*. *neocordata*, E = *D*. *dacunhai*, F = *D*. *milleri*, G = *D*. *sturtevanti*, H = *D*. *lehrmanae*, I = *D*. *lusaltans*, J = *D*. *prosaltans*, K = *D*. *septentriosaltans*, L = *D*. *pseudosaltans*, M = *D*. *nigrosaltans*, N = *D*. *austrosaltans*, O = *D*. *saltans*, P = *D*. *parasaltans*, Q = *D*. *willistoni*.

On the other hand, the Bayesian analyses with the concatenated mitochondrial-sequence dataset and Total Evidence dataset (morphological and molecular data) generated very similar trees resolving relationships among the subgroups with high supports (Figs 1B and 2). The *parasaltans* subgroup (represented by *D*. *parasaltans*) was placed as the sister to all the others in the *saltans* group (PP = 100%). The *saltans* subgroup was recovered as monophyletic (PP = 100%) and sister to a large clade consisting of the other three subgroups (PP = 100% for the mitochondrial dataset and PP = 97% for the Total Evidence dataset). Within the *saltans* subgroup clade, relationships among component species were less resolved, except for the close relationships ((*D*. *saltans*, *D*. *austrosaltans*), *D*. *nigrosaltans*) supported with PPs = 100%. Relationships among the three subgroups within the remaining clade were inferred as follows: [the *elliptica* + *cordata* subgroups (PP = 59% for the mitochondrial dataset and 75% for the Total Evidence dataset)] + [the *sturtevanti* subgroup (PP = 93%)]. Relationships among component species of the *elliptica* and *sturtevanti* subgroups were fully resolved: [[*D*. *emarginata* + *D*. *neoelliptica* (PP = 100%)] + [*D*. *neosaltans* (PP = 100%)]] in the *elliptica* subgroup; and [[*D*. *dacunhai* + *D*. *milleri* (PP = 100%)] + [*D*. *sturtevanti* + *D*. *lehrmanae* (PP = 97% for mitochondrial dataset and PP = 99% for Total Evidence dataset)]] in the *sturtevanti* subgroup.

The pairwise comparison of disparity index test and the composition distance indicated that the composition of *D*. *neosaltans* (*elliptica* subgroup) is significantly heterogeneous. Despite the conservative nature of the applied method, the null hypothesis that sequences have evolved with the same pattern of substitution for all the nucleotides was rejected in approximately 75% of the pairwise comparisons between *D*. *neosaltans* and the other species (S4 Table). The base composition bias by site found was higher for this species, especially in the third codon positions (S5 Table).

**Fig 3:**
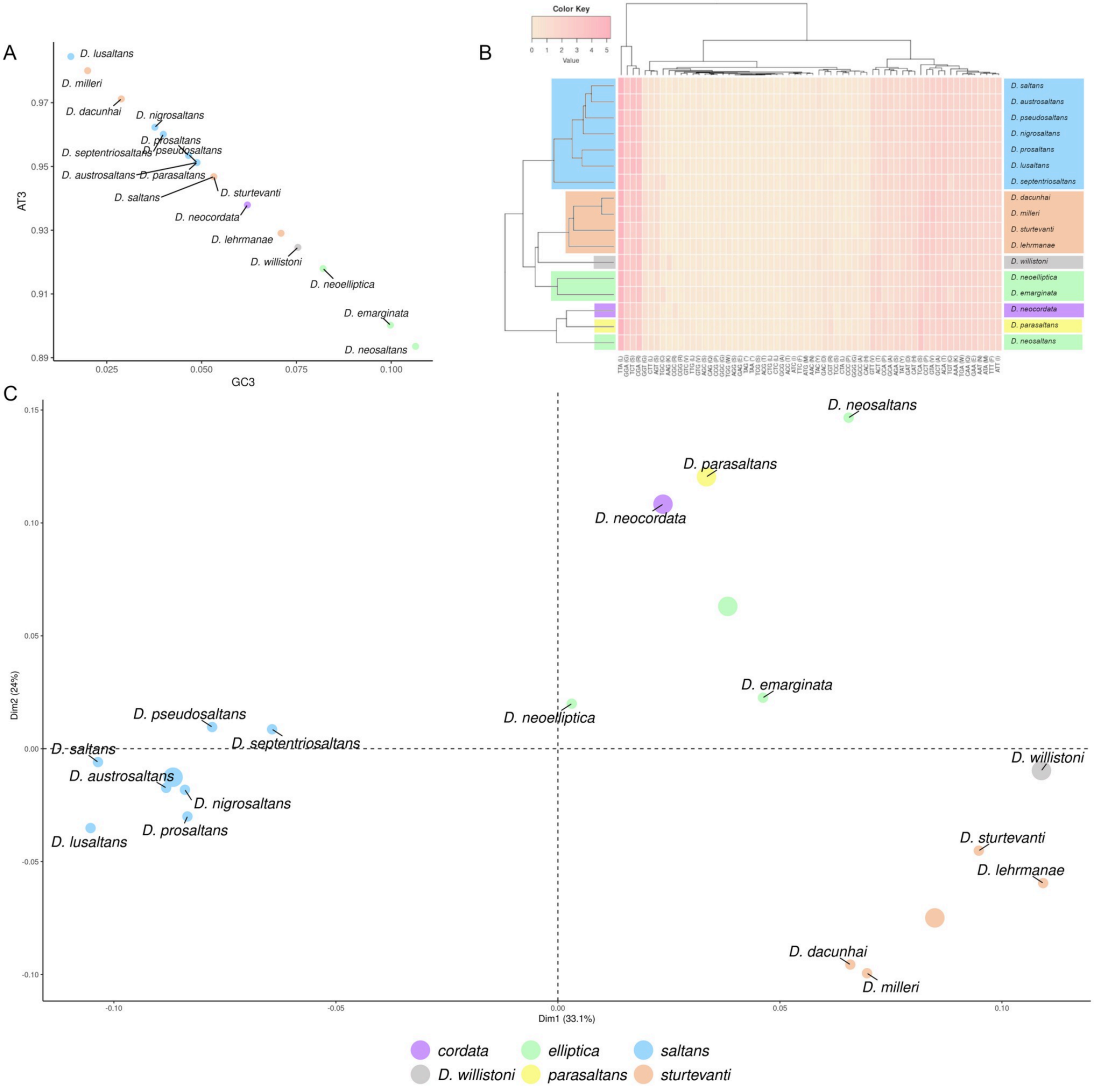
Codon usage bias pattern of mitochondrial genes *COI* and *COII* in the *saltans* group. (A) GC3 contents of *saltans*-group species. (B) Hierarchical clustering generated by heatmap. (C) Correspondence analysis of RSCU averages of 31 codons.

Aiming to verify the codon usage bias for the concatenated mitochondrial sequences of *COI* and *COII*, we calculated the GC content of the third codon position (GC3) for each species. Those results indicate that the mitochondrial region (*COI* and *COII*) is AT3 rich (varying from 89.4 to 98.5%; Fig 3A). The *elliptica* subgroup presented the highest GC3 (from 8.20 to 10.64%). The GC3 content varied most within the *sturtevanti* subgroup (from 2.00 to 7.10%), which the species *D*. *sturtevanti* and *D*. *lehrmanae* presented higher values of GC3 (5.3 and 7.1%) than *D*. *dacunhai* and *D*. *milleri* (2.9% and 2%, respectively). Within the *saltans* subgroup, the higher GC3 content was seen for the *D*. *saltans* (5.3%) and were particularly low for *D*. *lusaltans* (1.6%). Interestingly, the insular species (*D*. *lusaltans* and *D*. *milleri*) presented the lowest GC3. Through the heatmap the *saltans* and *sturtevanti* subgroups were recovered, while the *elliptica* subgroup was not because *D*. *neosaltans* was clustered with *D*. *neocordata* and *D*. *parasaltans* (Fig 3B). Similarly, the correspondence analysis also clustered of the *saltans* and *sturtevanti* subgroups (Fig 3C), which were separated by the first dimension of this analysis (see S1 Fig to check the contribution of each codon in each dimension). In the *elliptica* subgroup, the three species do not show similarity in the use of codons, which contributed the most to the first dimension, however considering the second dimension, *D*. *neoelliptica* and *D*. *emarginata* are very similar whereas *D*. *neosaltans* codon usage looks like *D*. *neocordata* and *D*. *parasaltans* (Fig 3C).

## Discussion

The combination of morphological and molecular markers proved to be very important for unraveling relationships among species within the *saltans* group and resulted in a robust phylogeny. In the analyses performed here, the studied species were classified into five lineages corresponding to the established subgroups [20], suggesting the reliability of the selected markers. *Drosophila parasaltans* is the only representative, in the present study, of the *parasaltans* subgroup and was recovered as a sister taxon of all the others in the *saltans* group, with high support, in the analysis of total evidence and molecules, corroborating with data from the literature [25, 51].

In our analysis with molecular markers, as well as in the analysis of total evidence, the *sturtevanti* subgroup is sister of *cordata*-*elliptica* clade. The close relationship between the *cordata* and *elliptica* subgroups corroborates several studies [24, 25, 28, 51]. It is interesting to demonstrate that in addition to the phylogenetic works mentioned above, the *cordata*-*elliptica* relationship was observed by Castro and Carareto [27] in a study with the P family of elements, in which the authors found a very rudimentary and divergent sequence of the same element in each *D*. *neocordata* (*cordata* subgroup) and *D*. *emarginata* (*elliptica* subgroup) species, suggesting proximity between these species and consequently between these subgroups.

The relationship among the species of *elliptica* subgroup was strongly supported in all analyses, and it was grouped in the same way, establishing two internal clades: one composed of *D*. *emarginata* and *D*. *neoelliptica* and another clade composed of *D*. *neosaltans*, close to the previous two. This is the first time that a phylogenetic study has been carried out with three of the four species included in this subgroup; other studies have only been performed with *D*. *emarginata* [24, 25, 28, 29, 51]. It is interesting to note that the relationships seen in the phylogenetic tree can also be observed in the morphological characters of these species, where the aedeagi of *D*. *emarginata* and *D*. *neoelliptica* are extremely similar, differing markedly from *D*. *neosaltans* [35]. The most striking features are found in the size and shape of aedeagus, in which aedeagus of *D*. *neosaltans* is smaller (~16% of body size, whereas for *D*. *emarginata* it is ~50% and *D*. *neoelliptica* ~30%) and has no sickle shape, the aedeagal apex is cylindrical and not hooked and forked and the pregonites are not fused to the end (Fig 2C) [35]. Furthermore, *D*. *neosaltans* differs from the other two species because apparently it does not present the structure of the epandrial ventral processes, and in the same place, it has only two small saliences, suggesting that this structure may be present in the ancestral species of the subgroup [35].

The relationships of species within the *sturtevanti* subgroup were recovered with good support. In all analyses, the species were similarly grouped, establishing two internal clades, one composed of *D*. *sturtevanti* and *D*. *lehrmanae* and the other composed of *D*. *dacunhai* and *D*. *milleri*. This cluster structure was also observed in the GC3 analysis, and it was observed by Madi-Ravazzi et al. [19], who analyzed the same species with four mitochondrial markers, and by Souza et al. [29], who used morphological characteristics without *D*. *lehrmanae* species. This subdivision can be visualized through the morphology, shape and size of the four aedeagus species, which are very similar, but there is a greater similarity of structures between *D*. *dacunhai* and *D*. *milleri*, such as the presence of pointed scales on the ventral postgonite and a groove in the upper part of it, whereas *D*. *sturtevanti* and *D*. *lehrmanae* present this structure without scales, smooth and thin (Fig 2E–2H) [35].

Furthermore, studies on reproductive isolation with four species (*D*. *dacunhai*, *D*. *magalhaesi*, *D*. *milleri* and *D*. *sturtevanti*) showed several degrees of isolation, from complete isolations to fertile crosses, and the presence of inseminated females in several crosses that did not produce progeny was observed, suggesting that these results may be related to the courtship behaviors and the similarity among the morphologies of the aedeagi [11, 19, 23]. So, although there is species specificity of male terminalia, the morphology of male terminalia among species in the *sturtevanti* subgroup is generally very similar, unlike what is seen in species in other subgroups of the *saltans* group. The question of why this similarity exists is unanswered.

The reconstruction of evolutionary relationships of the *saltans* subgroup has been considered particularly challenging, and inconsistencies have been reported for different molecular markers [24, 25], which were not solved by the analysis of morphological characters [28, 29]. To solve this problem, we analyzed all species of this subgroup; however, only the establishment of the sibling species *D*. *austrosaltans* and *D*. *saltans* resulting from the molecular and total evidence analyses of the present work was recovered. This last relationship is corroborated in studies by Bicudo [22] and Nascimento and Bicudo [52] carried out with chromosomal inversion and pattern of esterases, respectively. In addition, the morphological analysis showed that only the relationship between *D*. *nigrosaltans* and *D*. *pseudosaltans* was robustly recovered. This information is new in the *saltans* subgroup phylogeny, but it can be easily observed in the morphological characteristics of the aedeagus, as both species present elongated and curved back aedeagal apex, different from other species (Fig 2L and 2M) [35].

Previous studies found different topologies and polytomies for the *saltans* subgroup, which is considered the most recent divergence of the *saltans* group, having occurred approximately 4.5 million years ago [9, 24, 28]. Phylogenetic reconstruction among species of recent divergence can become much more complex due to three biological problems: segregation of polymorphisms that predate species divergence (incomplete lineage sorting), gene flow during and after speciation and intralocus recombination (hybridization) [53]. In fact, many recent studies have shown that these processes play an important role in the evolution of many taxa [54–56]. Gene flow among *saltans* subgroup species may still occur, and the species may continue to hybridize even at low frequencies [21]. This last study carried out a reproductive isolation experiment with seven species of the *saltans* subgroup and observed variable results, in which crosses between geographically distant species showed the production of fertile hybrids, even at low frequency [21]. Furthermore, in studies of chromosomal polymorphisms with this subgroup, Bicudo [22] found a common karyotype and a considerable degree of sequential homology for all species of the *saltans* subgroup. In addition, many other issues may lead to difficulties in constructing its phylogeny, such as rapid speciation rates relative to substitution rates, and heterogeneity in nucleotide composition biases [24, 28, 30, 31].

The codon usage bias analysis performed for the entire *saltans* group recovered low GC3 content in agreement with literature previews studies [57], which characterized the mitochondrial genes *COI*, *II* and *III* as sequences rich in adenine and cytosine, particularly at third codon position. The GC3 contents vary within the subgroups, and yet, in general, the subgroups were more similar within than among them. The reasons for the differences in degree of AT-content diversity among subgroups still are unclear, they could be associated with differences in the numbers of representative species among subgroups, differences in the ages of the subgroups, or nucleotide composition evolves faster in some subgroups. Furthermore, the codon usage for the genes *COI* and *COII* of the *saltans* group is similar to the pattern described for insects [57], with high values for the codons TTA (leucine), CGA (arginine), TCT (serine), GGA (glycine). The two clustering methods applied here recovery the *saltans* and *sturtevanti* subgroups but fail to recovery the *elliptica* subgroup due to CUB of *COI* and *COII* evolve in a heterogeneous way in *D*. *neosaltans*, the earliest to branch off among the *elliptica* subgroup. More mitochondrial genes should be evaluated to confirm if this is a mitogenome pattern, also evaluation of nuclear genes may be interesting to evaluate the mechanisms that may be shaping the CUB, particularly of *D*. *neosaltans*.

In general, the *saltans* group presents a variety of forms of aedeagus with great complexity, while the external morphology among the species of the group is very similar. This observation can be explained by Eberhard [58], who mentions that the male genitalia of animals subjected to internal fertilization evolve and diverge faster relative to the other morphological characters of the body [58, 59]. Consequently, the great diversity of morphological characters of male terminalia among genetically similar species is related to several complex phenomena driven by selective processes, mainly by sexual selection [58, 60, 61]. Furthermore, it is noteworthy that the rapid evolution of male genitalia is still sufficient to preserve a phylogenetic signal, which is especially useful in comparative and phylogenetic analyses among closely related species [59]. As an example, one study analyzed 41 phylogenetic articles from 11 different orders of arthropods, finding phylogenetically informative characters of the genitalia, suggesting that there is a rapid but ordered evolutionary rate [62]. Likewise, a comparison of 490 characters shows that adult terminalia characters present lower homoplasy than other organs [7].

In the current study, we use the greatest number of male terminalia morphological characters for the *saltans* group to date, demonstrating robust analysis associated with the most suitable mitochondrial genes for phylogenetic analysis of *COI* and *COII* [63, 64]. It is relevant to emphasize the importance of using different markers in phylogenetic analyses as soon as they present different mutation rates and coalescence times, which can provide different information with robust results, often complementary, and increase the accuracy of phylogenetic inferences about the processes involved [3, 4]. Therefore, the combination of these markers supported the *saltans* group as monophyletic and a new hypothesis of the relationship among species in the group, such as *parasaltans* subgroup as a sister taxon of the other species in the *saltans* group, followed by the formation of two clades: *saltans* subgroup clustered as sister of the large clade, consisting of the other three subgroups (*sturtevanti*, *cordata* and *elliptica*). Furthermore, the relationship of species within the *elliptica* and *sturtevanti* subgroups was well supported.

## Supporting information

S1 Fig Percent contribution of individual codons to the correspondence analysis of RSCU. The contributions of codons to the (A) first and (B) second dimension of correspondence analysis of 16 species of the *saltans* groups. The red dashed line indicates the expected average value if the contributions were uniform.
(TIF)

S1 Table Taxonomy and provenance information of the species included in this study. (DOCX)

S2 Table Primer sequences and PCR annealing temperatures. (DOC)

S3 Table RSCU averages of each codon in each species. Bold codons were used in correspondence analysis.
(XLSX)

S4 Table Results of pairwise tests for compositional homogeneity between the 17 studied species for all nucleotides, first, second, and third codon positions. P-values estimated from Monte Carlos test with 1,000 replicates are shown below the diagonal, significant P-values are highlighted.
(XLSX)

S5 Table Base composition test for the *saltans* species group. (XLSX)
