# Supplementary figures and images for: Phylogeny of Drosophila saltans group (Diptera: Drosophilidae) based on morphological and molecular evidence
Source: PLoS One. 2022 Apr 7;17(4):e0266710. doi: 10.1371/journal.pone.0266710 (PMC8989330; doi:10.1371/journal.pone.0266710)

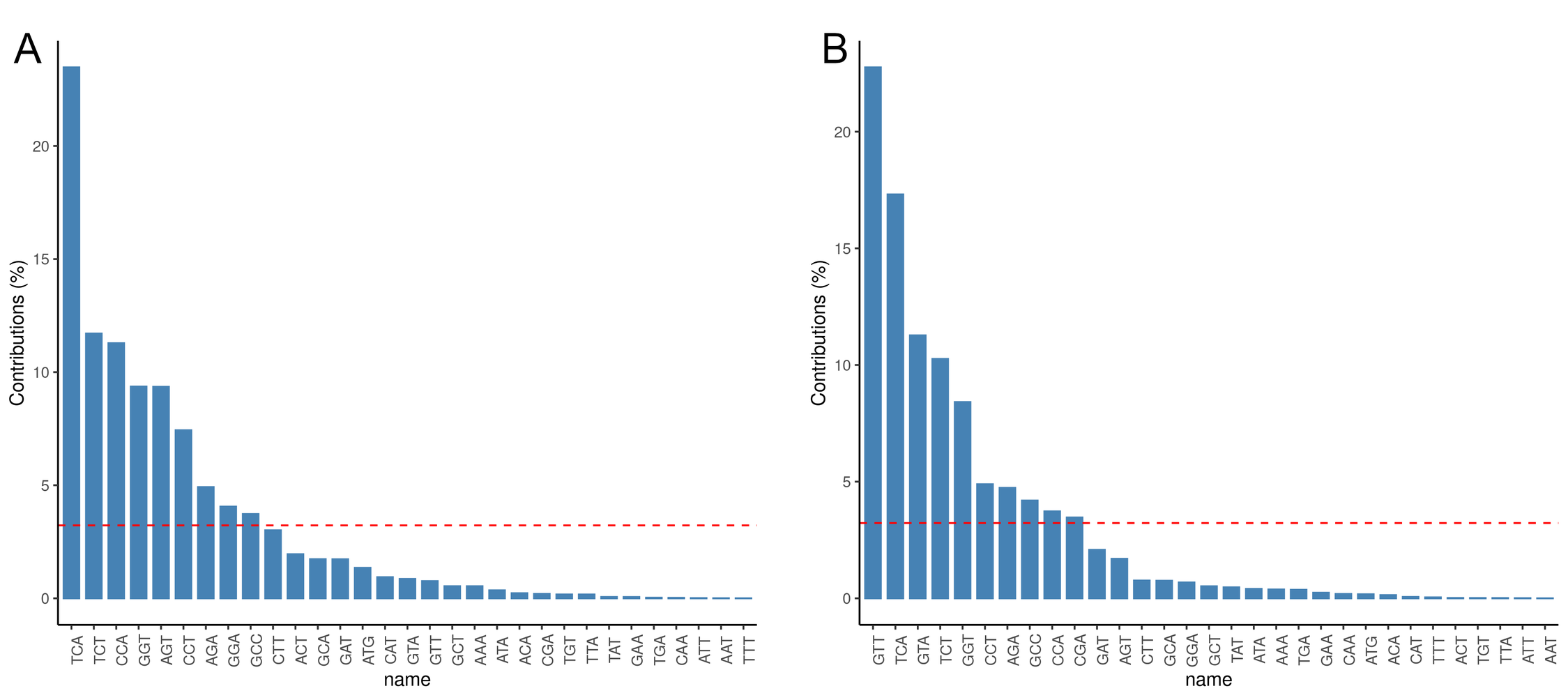

Supplement: S1 Fig — The contributions of codons to the (A) first and (B) second dimension of correspondence analysis of 16 species of the saltans groups. The red dashed line indicates the expected average value if the contributions were uniform. (TIF) [file pone.0266710.s001.tif]
