# Supplementary material for: Phylogeny of Drosophila saltans group (Diptera: Drosophilidae) based on morphological and molecular evidence
Source: PLoS One. 2022 Apr 7;17(4):e0266710. doi: 10.1371/journal.pone.0266710 (PMC8989330; doi:10.1371/journal.pone.0266710)
Supplement: S1 Table — (DOCX) [file pone.0266710.s002.docx]

**S1 Table**

| **Subgroups** | **Species** | **GenBank accession** | | **Strains** | **Geographical origin** |
| --- | --- | --- | --- | --- | --- |
|  |  | **COI** | **COII** |  |  |
| *saltans* | *D. saltans* | MZ700110 | MZ857172 | S4 | San José, Costa Rica |
|  | *D. prosaltans* | MZ700108 | MZ857170 | P1 | Picinguaba, SP, Brazil |
|  |  | - | - | P2 | Matão, SP, Brazil |
|  |  | - | - | P3 | Cantareira, SP, Brazil |
|  | *D. austrosaltans* | MZ700105 | MZ857168 | A2 | Nova Granada, SP, Brazil |
|  |  | - | - | A1 | Matão, SP, Brazil |
|  | *D. pseudosaltans* | MZ700109 | MZ857171 | PSE | Cantareira, SP, Brazil |
|  | *D. nigrosaltans* | MZ700107 | MZ857169 | NIG | Camp Nouragues, Inselberg, French Guiana |
|  |  | - | - | PLR | Camp Nouragues, Inselberg, French Guiana |
|  |  | - | - | A | Camp Nouragues, Inselberg, French Guiana |
|  | *D. septentriosaltans* | MZ700111 | MZ857173 | SEP | Camp Nouragues, Inselberg, French Guiana |
|  | *D. lusaltans* | MZ700106 | AF045090** | B44 (14045–0891.00)* | Petionville, Haiti |
| *sturtevanti* | *D. sturtevanti* | MZ700113 | MZ857174 | STV-1 | Matão, SP, Brazil |
|  |  | - | - | STV-2 | Aguaí, SC, Brazil |
|  |  | - | - | STV-3 | Ribeirão da Ilha, SC, Brazil |
|  | *D. milleri* | MZ700112 | MW820608.1** | MI-1 (14043–0861.00)* | El Yunque, Puerto Rico |
|  |  | - | - | MI-2 | Camp Nouragues, Inselberg, French Guiana |
|  | *D. dacunhai* | MW829428.1** | MW820607.1** | DAC | Petionville, Haiti |
|  | *D. lehrmanae* | MW829429.1** | MW820611.1** | STV-like | Camp Nouragues, Inselberg, French Guiana |
| *parasaltans* | *D. parasaltans* | MZ700118 | MZ857178 | B17–5 | Belém, PA, Brazil |
| *elliptica* | *D. emarginata* | MZ700114 | MZ857175 | JD | Vera Cruz, México |
|  | *D. neoelliptica* | MZ700115 | MZ857176 | NEO-1 | Cantareira, SP, Brazil |
|  |  | - | - | NEO-2 | Aguaí, SC, Brazil |
|  | *D. neosaltans* | MZ700116 | MZ857177 | H1 | Rio de Janeiro, RJ, Brazil |
|  |  | - | - | AG | Aguaí, SC, Brazil |
| *cordata* | *D. neocordata* | MZ700117 | AF045088** | CG | Campo Grande, MS, Brazil/ Minas Gerais, Brazil |
| *willistoni* | *D. willistoni* | JQ679116** | HQ110560** | - | - |

*Species obtained from UC San Diego Stock Center; **Sequence obtained from GenBank
