# Supplementary material for: Phylogeny of Drosophila saltans group (Diptera: Drosophilidae) based on morphological and molecular evidence
Source: PLoS One. 2022 Apr 7;17(4):e0266710. doi: 10.1371/journal.pone.0266710 (PMC8989330; doi:10.1371/journal.pone.0266710)
Supplement: S2 Table — (DOC) [file pone.0266710.s003.doc]

**S2 Table**

| Gene | Primer name | Sense | Primer | Sequence | Anneling temperature | Reference |
| --- | --- | --- | --- | --- | --- | --- |
| *COI* | TY-J-1460 | Forward | TY-J-1460 | 5’-TACAATCTATCGCCTAAACTTCAGCC-3’ | 56ºC | [59] |
|  | C-1-N-2191 | Reverse | C-1-N-2191 | 5’-CCCGGTAAAATTAAAATATAAACTTC-3’ |  | [61] |
| *COII* | TL2-J-3037 | Forward | TL2-J-3037 | 5’-ATGGCAGATTAGTGCAATGG-3’ | 58ºC | [62] |
|  | TK-N-3785 | Reverse | TK-N-3785 | 5' – GTTTAAGAGACCAGTACTTG-3' |  | [59] |
